# Supplementary material for: A plant chitinase controls cortical infection thread progression and nitrogen-fixing symbiosis
Source: eLife. 2018 Oct 4;7:e38874. doi: 10.7554/eLife.38874 (PMC6192697; doi:10.7554/eLife.38874)
Supplement: Figure 1—source data 1. [file elife-38874-fig1-data1.docx]

**Figure 1_source data 1**. Bacterial strains used in this study

| Strain | Relevant characteristics | Reference |
| --- | --- | --- |
| *Mesorhizobium loti* |  |  |
| R7A | Wild-type strain | ^34,42^ |
| *nodD1* | R7A *nodD1* deletion mutant | ^34^ |
| *nodD2* | R7A *nodD2* deletion mutant | ^34^ |
| *nolL* | R7A *nolL* deletion mutant | ^37^ |
| *nodZ* | R7A *nodZ* deletion mutant | ^37^ |
|  |  |  |
| *Agrobacterium* |  |  |
| AR1193 | For hairy root transformation | ^40^ |
