# Supplementary material for: A plant chitinase controls cortical infection thread progression and nitrogen-fixing symbiosis
Source: eLife. 2018 Oct 4;7:e38874. doi: 10.7554/eLife.38874 (PMC6192697; doi:10.7554/eLife.38874)
Supplement: Figure 1—source data 2. [file elife-38874-fig1-data2.docx]

**Figure 1_source data 2**. Primers used for RT-qPCR analyses

| Primer name | Sequence |
| --- | --- |
| ATP_F | CAATGTCGCCAAGGCCCATGGTG |
| ATP_R | AACACCACTCTCGATCATTTCTCTG |
| UBC_F | atgtgcattttaagacaggg |
| UBC_R | gaacgtagaagattgcctgaa |
| PP2A_F | GTAAATGCGTCTAAAGATAGGGTCC |
| PP2A_R | ACTAGACTGTAGTGCTTGAGAGGC |
| LB3_F | TGCTGCCACTCAACTCGTAG |
| LB3_R | AAGTCATCACTCCATTCGTCCG |
| SST1_F | TGGAATTCCAATCGTGGGCCA |
| SST1_R | CCAATTGCTATTCCTTCCGCC |
| MPK3_F | ATTGATCCCACCAAAAGAATCACAGTTGAA |
| MPK3_R | CCAATGCTTCCCTGTAGATCATCTC |
| WRKY29_F | GAATCTAAATATAAAAGAGGCAAG |
| WRKY29_R | ACCTGTAGTAGCTTCGAGGATAAGG |
| WRKY33_F | AATGAGGGTATATCAGCCCCTGG |
| WRKY33_R | GGGTGTGTGCATTTATAGTAACTCCTTG |
| N6_F | GCAATCCAGTTCTACAAGATCACA |
| N6_R | GGCTGCTCATTTCACAAGAACTCT |
| NPL_F | CCAAATGATGAAGAACACAAAGAGATCAC |
| NPL_R | CTTGACGCCCTAGCATATGTAGAAG |
| ERN1_F | CCACCCTTGTGCTCATTGTTCTG |
| ERN1_R | CCTACACTCCTCCCTCTCAAG |
