# Supplementary material for: A plant chitinase controls cortical infection thread progression and nitrogen-fixing symbiosis
Source: eLife. 2018 Oct 4;7:e38874. doi: 10.7554/eLife.38874 (PMC6192697; doi:10.7554/eLife.38874)
Supplement: Figure 3—source data 1. [file elife-38874-fig3-data1.docx]

**Figure 3_source data 1.** HPLC-MS analysis of Nod factor isolated after exposure to roots of wild-type Gifu or *chit5* mutants.

|  | Gifu | | | *chit5-1* | | | *chit5-2* | | | *chit5-3* | | |
| --- | --- | --- | --- | --- | --- | --- | --- | --- | --- | --- | --- | --- |
| Fraction | R1 | R2 | Avg | R1 | R2 | Avg | R1 | R2 | Avg | R1 | R2 | Avg |
| LCO-V | 82.3 | 75.5 | 78.9 | 92.7 | 87.6 | 90.15 | 88.3 | 90.3 | 89.3 | 90.3 | 89.7 | 90.0 |
| LCO-II | 17.7 | 24.5 | 21.1 | 7.3 | 12.4 | 9.85 | 11.7 | 9.7 | 10.7 | 9.7 | 10.3 | 10.0 |

R1 and R2 indicate biological replicates and Avg is the average of the replicates.
